# The Prognostic Value of the EASIX Score in Patients with Metastatic Pancreatic Cancer

**DOI:** 10.3390/diagnostics15141740

**Published:** 2025-07-09

**Authors:** Vahit Can Cavdar, Yalcin Gokmen, Mert Aric, Tugba Altunkaya, Cennet Gizem Erdem, Ilkay Gulturk, Cigdem Usul Afsar

**Affiliations:** 1 Department of Internal Medicine, Istanbul Training and Research Hospital, University of Health Sciences, Istanbul 34098, Turkey; yalcin_94@hotmail.com; 2 Department of Internal Medicine, Beylikduzu State Hospital, Istanbul 34500, Turkey; mert_aric@hotmail.com; 3 Department of Medical Oncology, Istanbul Training and Research Hospital, University of Health Sciences, Istanbul 34093, Turkey; tugbaltunkaya@gmail.com (T.A.); cgizemerdem@gmail.com (C.G.E.); gulturkilkay@gmail.com (I.G.); cigdemusul@yahoo.com (C.U.A.)

**Keywords:** pancreatic cancer, Endothelial Activation and Stress Index (EASIX), overall survival (OS)

## Abstract

**Background/Objectives:** Pancreatic cancer (PC) is an aggressive malignancy with a poor prognosis, frequently diagnosed at a metastatic stage. The identification of accessible, cost-effective prognostic biomarkers is critical for optimizing treatment strategies. The Endothelial Activation and Stress Index (EASIX), calculated using lactate dehydrogenase (LDH), creatinine, and platelet count, reflects endothelial dysfunction and has shown prognostic value in hematological cancers. However, its utility in metastatic PC remains unexplored. This study is the first to evaluate the prognostic significance of the EASIX in patients with metastatic PC receiving first-line FOLFIRINOX chemotherapy. **Methods:** This retrospective cohort study analyzed 204 patients diagnosed with metastatic pancreatic adenocarcinoma at Istanbul Training and Research Hospital between 2020 and 2025. All patients received FOLFIRINOX as first-line therapy. EASIX was calculated as LDH (U/L) × creatinine (mg/dL)/platelet count (10^9^/L). A cut-off value of 1.33 was used to stratify patients into low and high EASIX groups. Overall survival (OS) was assessed using Kaplan–Meier analysis and compared with the log-rank test. **Results:** The mean patient age was 63.0 ± 9.4 years; 61.8% were male. There were no significant differences in baseline characteristics between groups. Patients with EASIX ≥ 1.33 had significantly lower platelet counts and higher LDH and creatinine levels. Median OS was 14 months for EASIX < 1.33 and 8 months for EASIX ≥ 1.33 (*p* < 0.001). **Conclusions:** EASIX is a simple, inexpensive prognostic marker associated with overall survival in metastatic PC. Its integration into clinical practice may facilitate early risk stratification. Further prospective studies are needed to confirm its prognostic utility.

## 1. Introduction

PC is one of the most aggressive solid organ tumors, characterized by its rapid spread among cancers and holds the fourth position among the primary causes of cancer-related mortality worldwide [1]. The difficulty in discussing curative treatments for pancreatic cancer arises from the fact that, even at the time of diagnosis, more than 80% of cases are already metastatic [2]. Therefore, the role of surgery in pancreatic cancers is limited to being a treatment option for only a limited proportion of patients. Given that the majority of cases are identified during metastatic or late-stage disease of PC, surgical treatment is not an option for them, and they are generally treated with systemic chemotherapy. In recent years, the incorporation of newly developed biomarkers and therapeutic agents has led to significant progress in the management of metastatic pancreatic cancer.

Although we have become more aware and attentive regarding the risk factors and screening of pancreatic cancer, there is still a need to utilize biomarkers that can predict the prognosis and the course of the disease from the time of diagnosis. The use of such markers is considered essential for better disease management and improving survival outcomes.

Numerous inflammation-based scoring systems have been introduced in the literature through various studies for predicting prognosis in PC, and they have been shown to have predictive value at the time of initial diagnosis. Among these, the Naples Prognostic Score (NPS) has recently emerged as a comprehensive scoring system that combines inflammatory and nutritional parameters, providing a comprehensive assessment of the patient’s status [3,4]. Additionally, the Glasgow Prognostic Score (GPS) [5] and the Modified Glasgow Prognostic Score (mGPS) [6] are widely used, both based on serum C-reactive protein and albumin levels, reflecting the systemic inflammatory response and nutritional status. Other inflammation-based indices such as the neutrophil-to-lymphocyte ratio (NLR) [7], platelet-to-lymphocyte ratio (PLR) [8], and Systemic Immune-Inflammation Index (SII) [9] have also gained attention due to their easy accessibility and prognostic significance. These indices provide indirect information about the balance between the host’s immune response and tumor-promoting inflammation. Furthermore, the Prognostic Nutritional Index (PNI) [10], which incorporates serum albumin and lymphocyte count, is another important tool that reflects both nutritional and immunological status and has also been associated with survival outcomes in PC patients.

The Endothelial Activation and Stress Index (EASIX) score is a very practical, simple, and cost-effective scoring system that can be easily computed using the following routine laboratory parameters: serum creatinine, serum lactate dehydrogenase (LDH), and platelet count. The formula for calculating the EASIX score is: LDH (U/L) × creatinine (mg/dL)/platelet count (10^9^/L). The EASIX score was initially developed as a predictor of endothelial dysfunction and related complications, particularly in patients undergoing allogeneic hematopoietic stem cell transplantation. Endothelial damage plays a crucial role in the development of transplant-associated complications such as graft-versus-host disease (GVHD) and post-transplant mortality. In hematology, the EASIX score has been established as a prognostic marker for predicting survival after allogeneic stem cell transplantation [11], estimating the risk of acute GVHD [12], and assessing treatment outcomes in diffuse large B-cell lymphoma, the most common subtype of non-Hodgkin lymphoma [13]. Recently, its prognostic relevance has also been demonstrated in solid tumors, including metastatic cancers, as it indirectly reflects systemic inflammation, endothelial dysfunction, and microvascular damage—factors known to contribute to tumor progression and poor survival. Due to its simplicity, reproducibility, and reliance on widely available laboratory tests, the EASIX score has emerged as a reliable tool for risk stratification in various oncological settings.

Our study sought to assess the prognostic value of the EASIX score and its predictive ability for OS in patients with metastatic PC receiving first-line chemotherapy. Based on existing literature, this investigation represents the initial effort to analyze the prognostic relevance of the EASIX score specifically in this patient population.

## 2. Material and Methods

### 2.1. Patient Group

Clinical data were obtained from Istanbul Training and Research Hospital, Istanbul, Türkiye. We conducted a retrospective analysis on 288 consecutive individuals diagnosed with metastatic PC who had not received prior systemic chemotherapy and were treated with first-line chemotherapy between 2020 and 2025. Only patients with histologically confirmed pancreatic adenocarcinoma and measurable metastatic lesions, as defined by the Response Evaluation Criteria in Solid Tumors (RECIST), were eligible for inclusion. To minimize variability related to treatment outcomes and maintain consistency across the cohort, the study was limited to patients who received FOLFIRINOX as their initial chemotherapy regimen.

The study excluded patients presenting with any concurrent infectious disease, those with pre-existing chronic kidney disease, those who lacked key laboratory results (such as complete blood count, serum creatinine, or LDH levels) within 2 weeks prior to starting first-line chemotherapy, or who had received anti-cancer therapies other than the specified chemotherapy protocol. Around 84 individuals were not included due to incomplete data or failure to meet the study’s inclusion criteria. Additionally, those who did not attend scheduled follow-up visits, had incomplete laboratory records, or continued their treatment at other healthcare institutions were also excluded. As a result, 204 patients were eligible and included in the final evaluation.

### 2.2. Data Collection

The evaluated parameters included: patients’ gender, age, ECOG performance scores, white blood cell counts, hemoglobin levels, platelet counts, absolute neutrophil, lymphocyte, and monocyte counts. Based on these values, NLR (neutrophil-to-lymphocyte ratio) and LMR (lymphocyte-to-monocyte ratio) were calculated. Additional laboratory parameters such as urea, creatinine, and LDH levels were also recorded. Furthermore, metastatic sites at the time of diagnosis, EASIX scores, and OS durations were documented. OS was measured as the period commencing at the initiation of first-line chemotherapy and continuing up to the time of death for each patient.

The subjects were divided into two groups based on an EASIX cut-off score of 1.33. This cut-off value was adopted from the study conducted by Park S. et al., which evaluated the prognostic value of the EASIX score in patients with diffuse large B-cell lymphoma [13]. In that study, the threshold of 1.33 was determined using receiver operating characteristic (ROC) curve analysis for survival stratification. Currently, there is no established EASIX cut-off value specific to pancreatic cancer in the literature. Therefore, we utilized this previously defined cut-off as a reference point for our study to provide an initial framework for risk stratification in this patient population.

### 2.3. Statistical Analysis

Summary statistics are presented as mean, standard deviation, median, minimum, maximum, frequency, and percentage. The distribution of the variables was assessed using the Kolmogorov–Smirnov and Shapiro–Wilk tests. For the comparison of quantitative independent variables with normal distribution, the independent samples *t*-test was used. For non-normally distributed quantitative variables, the Mann–Whitney U test was applied. Categorical variables were compared using the chi-square test. OS of the cases was estimated using the Kaplan–Meier method, and survival differences between EASIX score groups were compared using the log-rank test. OS estimates were presented as median values with 95% confidence intervals. All analyses were carried out with IBM SPSS Statistics for Windows, version 28.0.

## 3. Results

Patients’ mean age at inclusion was 63.0 ± 9.4 years. Males accounted for 61.8% (*n* = 126) of the patients, with females making up 38.2% (*n* = 78). The ECOG performance status at the time of diagnosis, baseline complete blood count and biochemical parameters, initial EASIX scores, and presence of liver, lymph node, and bone metastases at diagnosis are presented in Table 1.

No significant variations were observed between patients with EASIX scores < 1.33 and ≥1.33 in terms of age, sex distribution, or ECOG performance status (*p* > 0.05) (Table 2).

Statistical analysis showed no significant differences between the groups with EASIX scores < 1.33 and ≥1.33 in terms of LMR, NLR, WBC, and hemoglobin levels (*p* > 0.05). The platelet count was significantly lower in the group with EASIX scores ≥ 1.33 compared to those with scores < 1.33 (*p* = 0.000). However, this difference is essentially expected due to the mathematical structure of the EASIX formula, which inversely includes platelet count. Therefore, while the statistical difference in platelet counts supports the calculation formula, it may not provide independent clinical insight on its own. Notably, the clinical relevance is reflected in the significantly higher levels of urea, creatinine, and LDH in the ≥1.33 EASIX group (*p* < 0.05), which may indicate increased endothelial dysfunction, organ stress, and systemic disease burden in these patients.

The groups did not differ significantly in terms of liver, lymph node, or bone metastases (*p* > 0.05) (Table 2).

**Figure 1:**
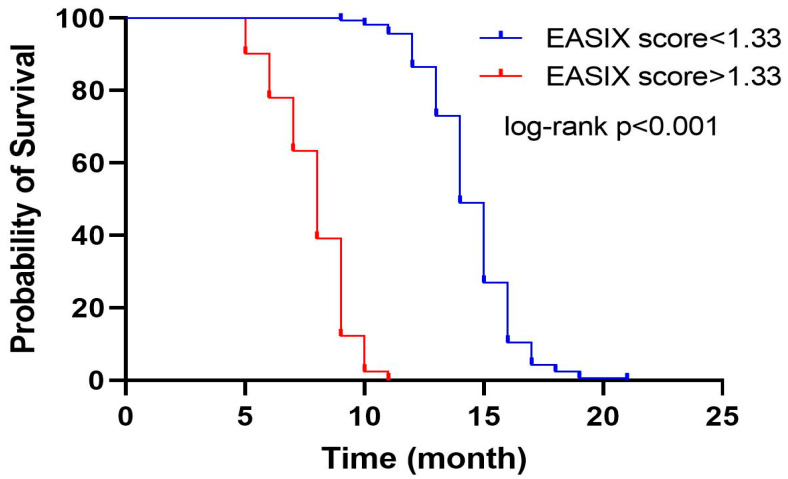
Kaplan–Meier overall survival curves of the patients according to EASIX score groups. EASIX: Endothelial Activation and Stress Index.

OS durations of the patients were calculated using Kaplan–Meier survival curves based on EASIX scores, and survival outcomes were compared by means of the log-rank test. The median OS for all cases was determined to be 14 months (95% confidence interval [CI]: 13.65–14.35). EASIX score was found to have a statistically significant impact on survival duration (Figure 1). Patients with an EASIX score < 1.33 had a median survival of 14 months (95% CI: 13.66–14.33), whereas those with an EASIX score > 1.33 had a median survival of 8 months (95% CI: 7.39–8.61), and this difference was statistically meaningful (*p* < 0.001) (Table 3).

## 4. Discussion

PC becomes more frequent and deadly among both males and females as they age, with the highest diagnosis rate in those over 70 years old [14]. In our study population, PC was observed in patients as young as 34 and as old as 86, with a mean age of 63 years. PC is more commonly observed in men than in women [15]. Similarly, in our study, 61.8% of the patients were male and 38.2% were female.

Multiple research efforts have explored the link between performance status and survival in PC; however, the results have been conflicting. While some studies have found that poor baseline performance status is strongly linked to poorer survival across all stages of PC, another study reported that although performance status had an impact on survival in univariate analysis, its prognostic significance was lost in multivariate analysis [16,17]. Similarly, our study found no significant difference in performance status between the low and high EASIX score groups, which indicates that another factor influencing mortality was likely not involved.

When the results of our study are examined, it was observed that platelet counts were significantly lower in the group with a high EASIX score, supporting existing literature that highlights the association between thrombocytopenia and PC and its relationship with poor prognostic factors. Platelets play not only a role in coagulation but also take on critical roles in cancer pathophysiology. Studies have shown that platelets contribute to tumorigenesis and metastasis through complex interactions with cancer cells. Extracellular vesicles released by platelets facilitate intercellular communication within the tumor microenvironment and promote tumor progression [18]. One of the proposed mechanisms of thrombocytopenia in solid tumors is related to polymorphisms or mutations in certain transcription factors and cytokines involved in megakaryocytic maturation, or may result from the adverse effects of therapies [19]. Studies have reported that white blood cell count is an independent prognostic factor for OS in patients with PC. Although low hemoglobin levels have been associated with poorer OS, their significance as an independent prognostic marker has not been confirmed in multivariate analyses [20]. Our study revealed no significant variations in WBC count or hemoglobin levels between patients with low versus high EASIX scores, and our findings did not support the associations reported in the literature.

LDH concentrations correlate with the extent of tumor burden and may serve as indicators of tumor progression and invasive capacity. LDH is a key enzyme in anaerobic glycolysis, and its elevation often indicates the metabolic reprogramming of cancer cells known as the Warburg effect, where tumor cells preferentially utilize anaerobic pathways despite the presence of adequate oxygen [21].

This adaptation facilitates rapid tumor growth, promotes angiogenesis, and contributes to chemotherapy resistance [22].

Several studies have shown that high serum LDH levels are poor prognostic factors in cancer patients. According to research carried out by Tas et al., elevated LDH levels were significantly associated with tumor burden and were reported to reflect the tumor’s proliferative and invasive potential [23]. Similarly, Stocken et al. demonstrated that LDH is an independent prognostic marker in patients with advanced-stage PC [24]. In another study by Haas et al., pretreatment LDH levels showed a strong association with time to progression in univariate analysis [25]. Consistent with these findings, our study showed that patients in the high EASIX score group had significantly elevated LDH levels. Given the established association between high EASIX scores and reduced OS, it can be inferred that elevated LDH may also serve as an indicator of poor prognosis in patients with metastatic PC.

As one of the components of the EASIX score, serum creatinine levels were observed to be significantly higher in patients with high EASIX scores in our study. It is considered that cancer-related conditions, such as loss of appetite, reduced oral intake, nausea, and vomiting, may have contributed to decreased hydration in these patients, potentially leading to renal impairment. Beyond these contributing factors, cancer-related systemic inflammation and endothelial dysfunction can also compromise renal perfusion, leading to acute or chronic kidney injury [26].

In cancer patients, the tumor microenvironment triggers a persistent systemic inflammatory response characterized by elevated cytokines such as IL-6, TNF-α, and IL-1β, which can impair renal vascular tone and lead to renal hypoperfusion and structural kidney damage over time. Sustained inflammation promotes glomerular and interstitial fibrosis, increasing the risk of chronic kidney disease [27]

Additionally, cancer-related endothelial dysfunction, which is partly driven by pro-inflammatory cytokines and oxidative stress, reduces nitric oxide bioavailability, leading to microvascular constriction and impaired renal microcirculation [28].

Cancer-associated hypercoagulability further contributes to this process by promoting microthrombus formation in glomerular and peritubular capillaries, exacerbating renal perfusion defects [29].

Consequently, it is suggested that the group with renal dysfunction may have a poorer prognosis. A review of the literature reveals that some studies have identified BUN as a standalone predictor of outcomes in PC, whereas serum creatinine has not demonstrated the same prognostic significance [20].

Pancreatic cancer is marked by early spread through the lymphatic system, swift disease progression, and unfavorable prognosis [30]. When diagnosed, the majority of patients already exhibit lymph node metastases [31]. The presence of positive lymph nodes is independently linked with a poorer prognosis in patients with metastatic PC [32]. In their study on PC, Riediger and colleagues reported that the lymph node ratio was the most powerful indicator for predicting survival among 204 patients who underwent surgical resection. They further suggested that routine calculation of the lymph node ratio could be beneficial for individualized prognostic assessment as well as for guiding decisions regarding adjuvant therapy [33]. In our study, 182 patients (89.2%) presented with lymph node metastasis at diagnosis, establishing it as the predominant site for metastatic spread.

The liver was identified as the second-most frequent site of metastasis, observed in 87 patients (42.6%). The metastasis of pancreatic cancer to the liver is a complex, multistep process. A marked similarity exists between the primary tumor and the metastatic lesions in the liver; while primary tumor cells exhibit pronounced invasive characteristics, metastatic cells demonstrate a high proliferative capacity. The adaptation of tumor cells to, and their interaction with, the surrounding microsystem facilitates the colonization and subsequent proliferation of PC cells within the liver [34]. Genomic analyses have revealed a high degree of genetic similarity between in situ pancreatic cancer lesions and liver metastases. Notably, mutations in PC-associated driver genes such as KRAS, TP53, and SMAD4 were found to be nearly identical in both tumor sites [35]. Metastatic spread to the liver is commonly observed in pancreatic adenocarcinoma. Over half of patients diagnosed with PC present with liver metastases at the time of diagnosis, and this condition is considered to indicate a poor prognosis [36,37]. Within our research, this rate was found to be 42.6%, aligning with previously published data.

In our study, bone metastasis was identified in 32 patients, corresponding to a rate of 15.7%, and was considered the least common site of metastasis. These outcomes correspond to those reported in prior studies, which suggest that the incidence of bone metastases in PC ranges between 5% and 20% [38,39].

In our study, no significant difference was detected between patients with high and low EASIX scores in terms of metastatic sites. It is thought that the high metastatic rate of PC at the time of diagnosis results in a substantial proportion of patients presenting with metastasis, regardless of whether the EASIX score is high or low. The difficulty in identifying patients at a stage where curative surgery is feasible contributes to PC being one of the deadliest cancer types worldwide.

Research conducted by Gasiorowska et al. demonstrated that patients with PC had significantly elevated plasma levels of adiponectin, TNF-α, and IL-6 compared to healthy individuals. Additionally, levels of endothelial dysfunction markers such as ICAM and VCAM were found to be increased in PC. These findings suggest enhanced endothelial activation and inflammation in individuals with PC [40]. The EASIX score, which is calculated using serum creatinine, LDH, and platelet count, serves as a marker of endothelial dysfunction. In our study, we examined the impact of the EASIX score on mortality in patients with metastatic PC and found that those with high EASIX scores had significantly higher mortality rates. These findings highlight the prognostic significance of endothelial distress and dysfunction in PC.

Following studies demonstrating the use of the EASIX score as a prognostic marker in hematological malignancies, several publications have investigated its prognostic and survival predictive value in solid organ tumors as well. In 2022, a study by Go S-I et al. demonstrated that the EASIX score is a predictive biomarker for survival in small cell lung cancer. The study showed that a high EASIX score was associated with poorer outcomes in terms of both progression-free survival and OS [41]. According to the 2022 research conducted by Gu JS et al., which included 627 patients, high EASIX scores were associated with poorer recurrence-free survival and OS in individuals with upper tract urothelial carcinoma [42]. Martin SD et al. performed a study in 2023 reporting that the EASIX score may serve as a prognostic biomarker in patients with metastatic renal cell carcinoma receiving immune checkpoint inhibitor therapy. The study revealed that patients with higher EASIX scores were linked with poorer prognosis and lower treatment response rates [43].

To date, no studies in the literature have investigated the impact of the EASIX score on mortality in pancreatic cancer, highlighting the significance of our study. Given that the EASIX score can be calculated using a simple complete blood count and basic biochemical tests in an outpatient setting, we believe it is a practical, easy, inexpensive, and applicable method. With further studies, it is likely to be widely adopted in the future for cancers and diseases in which endothelial dysfunction and stress play a role in the pathogenesis.

## 5. Conclusions

The findings of our study demonstrate that the EASIX score is a significant and independent prognostic marker for OS in subjects with metastatic PC administered first-line chemotherapy. Patients with elevated EASIX scores exhibited markedly shorter survival times and higher mortality rates compared to those with lower scores. Given its simplicity, cost-effectiveness, and ease of calculation using routinely available laboratory parameters, the EASIX score may serve as a valuable tool in routine clinical practice for early risk stratification. Furthermore, our results underscore the role of endothelial dysfunction in the pathophysiology and prognosis of PC. Further prospective studies are needed to validate the prognostic utility of the EASIX score and explore its potential integration into standard oncologic risk assessment protocols.

## Figures and Tables

**Table 1:** Overview of clinical, laboratory, and metastatic features of the patients.

		Min–Max			Median	Mean ± sd/*n*-%		
Age (year)		34.0	–	86.0	63.5	63.0	±	9.4
Gender	Male					126		61.8%
	Female					78		38.2%
ECOG Score	0					62		30.4%
	I					119		58.3%
	II					17		8.3%
	III					6		2.9%
Lymphocyte-to-Monocyte Ratio		0.3	–	13.0	2.8	3.3	±	2.0
Neutrophil-to-Lymphocyte Ratio		0.7	–	32.5	2.9	4.4	±	4.4
White Blood Cell Count (×10^3^/µL)		1.0	–	162.3	7.5	11.4	±	19.9
Hemoglobin (g/dL)		7.2	–	18.7	12.1	12.0	±	1.9
Platelet Count (×10^3^/µL)		45.0	–	919.0	263.0	269.2	±	118.7
Urea (mg/dL)		7.0	–	166.0	32.0	40.7	±	26.0
Creatinine (mg/dL)		0.26	–	1.78	0.72	0.79	±	0.31
Lactate Dehydrogenase (U/L)		101.0	–	615.0	213.0	235.5	±	94.8
EASIX Score		0.07	–	7.46	0.61	0.84	±	0.93
Liver Metastasis	(−)					117		57.4%
	(+)					87		42.6%
Lymph Node Metastasis	(−)					22		10.8%
	(+)					182		89.2%
Bone Metastasis	(−)					172		84.3%
	(+)					32		15.7%

ECOG: Eastern Cooperative Oncology Group; EASIX: Endothelial Activation and Stress Index; Min: Minimum; Max: Maximum; sd: Standard deviation.

**Table 2:** Clinical and laboratory characteristics of metastatic pancreatic cancer patients according to EASIX score.

		EASIX Score < 1.3 (*n*: 163)				EASIX Score ≥ 1.3 (*n*: 41)				*p*	
		Mean ± sd/*n*-%			Median	Mean ± sd/*n*-%			Median		
Age		62.8	±	9.4	63.0	63.9	±	9.5	66.0	0.437	^m^
Gender	Male	98		60.1%		28		68.3%		0.336	^X2^
	Female	65		39.9%		13		31.7%			
ECOG Score	0	53		32.5%		9		22.0%		0.189	^X2^
	I	92		56.4%		27		65.9%			
	II	12		7.4%		5		12.2%			
	III	6		3.7%		0		0.0%			
Lymphocyte-to-Monocyte Ratio		3.3	±	2.0	2.8	3.3	±	2.0	2.9	0.936	^m^
Neutrophil-to-Lymphocyte Ratio		4.3	±	4.0	3.0	4.8	±	5.9	2.7	0.605	^m^
White Blood Cell Count (×10^3^/µL)		12.2	±	22.0	7.5	8.1	±	4.4	7.3	0.454	^m^
Hemoglobin (g/dL)		12.0	±	1.9	12.0	12.1	±	2.0	12.1	0.640	^t^
Platelet Count (×10^3^/µL)		284.4	±	121.0	269.0	208.4	±	86.4	200.0	***0.000***	^m^
Urea (mg/dL)		37.6	±	24.0	28.0	53.2	±	29.8	42.0	***0.000***	^m^
Creatinine (mg/dL)		0.71	±	0.27	0.64	1.08	±	0.30	1.04	***0.000***	^m^
Lactate Dehydrogenase (U/L)		217.3	±	84.0	204.0	307.9	±	101.6	304.0	***0.000***	^m^
Liver Metastasis	(−)	94		57.7%		23		56.1%		0.856	^X2^
	(+)	69		42.3%		18		43.9%			
Lymph Node Metastasis	(−)	17		10.4%		5		12.2%		0.745	^X2^
	(+)	146		89.6%		36		87.8%			
Bone Metastasis	(−)	138		84.7%		34		82.9%		0.785	^X2^

^t^ Independent Samples *t* test/^m^ Mann–Whitney u test/^X2^ Chi-square test. ECOG: Eastern Cooperative Oncology Group; Min: Minimum; Max: Maximum; sd: Standard deviation.

**Table 3:** Overall survival estimates of the patients using the Kaplan–Meier method and comparison between EASIX score groups.

	OS (Month) Median (95%CI)	*p*
EASIX score < 1.33	14 (13.66–14.33)	**<0.001**
EASIX score > 1.33	8 (7.39–8.61)	
Overall	14 (13.65–14.35)	

The *p*-value was obtained from the log-rank test. OS refers to overall survival.

## Data Availability

The data produced and/or examined in this study cannot be shared publicly due to patient confidentiality and institutional restrictions but may be available from the corresponding author upon reasonable request and with permission from Istanbul Training and Research Hospital.
